# Prediction of Frailty and Dementia Using Oral Health Impact Profile from a Population-Based Survey

**DOI:** 10.3390/ijerph17061997

**Published:** 2020-03-18

**Authors:** Chi-Jung Tai, Jen-Hao Chen, Tzyy-Guey Tseng, Yi-Ting Lin, Yu-Han Hsiao, Meng-Chih Lee, Yi-Hsin Yang

**Affiliations:** 1 Department of Family Medicine, Pingtung Hospital, Ministry of Health and Welfare, Pingtung 90054, Taiwan; taichijung@gmail.com; 2 Graduate Institute of Natural Products, College of Pharmacy, Kaohsiung Medical University, Kaohsiung 80708, Taiwan; 3 School of Dentistry, College of Dental Medicine, Kaohsiung Medical University, Kaohsiung 80708, Taiwan; jehach@kmu.edu.tw; 4 Department of Dentistry, Kaohsiung Municipal Hsiao-Kang Hospital, Kaohsiung 812, Taiwan; 5 Department of Family Medicine, Kaohsiung Medical University Hospital, Kaohsiung Medical University, Kaohsiung 80708, Taiwan; tzyyguey@gmail.com (T.-G.T.); emilyhei@gmail.com (Y.-T.L.); 6 Faculty of Medicine, College of Medicine, Kaohsiung Medical University, Kaohsiung 80708, Taiwan; 7 Department of Medical Sciences, Molecular Epidemiology, Uppsala University, Uppsala SE-75185, Sweden; 8 Department of Family Medicine, Taichung Hospital, Ministry of Health and Welfare, Taichung 40343, Taiwan; phoebe01026@gmail.com; 9 Department of Public Health, Chung Shan Medical University, Taichung 40201, Taiwan; 10 College of Management, Chaoyang University of Technology, Taichung 41331, Taiwan; 11 Institute of Population Health Sciences, National Health Research Institutes, Miaoli 35053, Taiwan; 12 School of Pharmacy, College of Pharmacy, Kaohsiung Medical University, Kaohsiung 80708, Taiwan; 13 National Institute of Cancer Research, National Health Research Institutes, Tainan 70456, Taiwan

**Keywords:** oral frailty, dentition, gustatory system, cognitive impairment

## Abstract

Oral health and dentition have been associated with cognitive ability and frailty, but an applicable screening tool has not yet been developed. This study aimed to establish risk prediction models for dementia and frailty. A sample of 2905 community-dwelling older adults aged ≥58 years using the Taiwan Longitudinal Study on Aging (TLSA) survey was adapted and analyzed for this study. Risk scores were estimated by stepwise logistic regression. In models adjusted for covariates, increased age, female sex, no dental prosthesis (adjusted Odds ratio [adjOR], 1.61; 95% confidence interval [CI], 1.11–2.35), diabetes mellitus, chronic kidney disease, and an increased Oral Health Impact Profile (OHIP)-7T Q3 score (adjOR, 1.33; 95% CI, 1.19–1.49) were all significantly associated with frailty. In addition to these factors, an inability to self-report height or weight (adjOR, 4.52; 95% CI, 3.52–5.81) and an increased OHIP-7T Q7 score (adjOR, 1.21; 95% CI, 1.06–1.37) were significantly associated with dementia. The cut-off points of the risk scores for frailty and dementia were 80 (sensitivity, 80.0%; specificity, 81.2%) and 77 (sensitivity, 83.4%; specificity, 71.5%), respectively. The findings highlighted a number of composite risk factors of frailty and dementia. Importantly, the developed prediction models were easily applicable to screen for frailty and dementia in communities or dental clinics.

## 1. Introduction

The growing aging population has become an important health issue in many countries. To ensure good health in elderly people, any aging related physiologic or psychologic changes such as functional decline, cognitive impairment, and dentition changes would need to be prevented and managed [1]. Recently, many clinicians have paid attention to frailty, which is a common syndrome in older adults that carries an increased risk of poor health outcomes including falls, incident disability, hospitalization, and mortality [2,3,4]. Frailty is generally associated with a loss of skeletal muscle mass, diminished muscle strength, clinical comorbidities and reduced physical performance, which together form a vicious circle of disability [5]. The estimated prevalence of frailty among older adults ranges between 4.1% and 11.5% [6]. Concurrently, dementia, the decline in memory and other cognitive functions, is also a common health problem with a prevalence of 8.8%–11.6% in the United States, and of 5.14%–18.6% in Asia including Japan, China, Korea, and Philippine [7,8,9,10,11,12]. Dementia patients lose their independent function, which generally causes a large socioeconomic impact on patients, families, and government programs [13]. While frailty and dementia have become challenging health issues in aging populations, it is important to identify modifiable risk factors for the early intervention and consequently reduce the incidence of these diseases.

The concept of oral health-related quality of life (OHRQoL) has been adapted in many studies to evaluate the effect of oral disorders and dental treatments [14]. The most commonly used OHRQoL instrument is the Oral Health Impact Profile (OHIP) [15], which is based on Locker’s conceptual model. This model of OHRQoL describes that oral conditions and symptoms may lead to functional limitations, and hence physical pain and psychological discomfort [16]. Therefore, the consequent disability (physical, psychological, or social) can lead to handicap of quality of life. The OHIP initially constituted a set of 49 items (OHIP-49) with seven dimensions (functional limitation, physical and psychological discomfort, physical, psychological and social disability, and handicap) [15], and later a short version of 14 items (OHIP-14) was also generated to be implemented in large-scale community survey [17].

Oral health problems including tooth loss, the use of dentures, and reduced masticatory function are widely prevalent in elderly adults. Notably, growing evidence has suggested that poor oral health is significantly associated with frailty and dementia [18,19]. A systemic review also concluded that mastication has a positive association with cognitive function among older adults [20]. Specifically, one study showed that frail older adults had a lower number of remaining teeth and poorer oral hygiene compared to robust and pre-frail elderly adults [21]. A previous study also showed that older adults with oral pain or chewing problems had significantly higher risk of being frail [22]. In addition, reduced masticatory function has been associated with lower cognitive function [23].

Although oral health was found to have a significant relationship with frailty and dementia in previous studies, it had received little attention from public policy makers. Moreover, an applicable screening tool of frailty and dementia has not yet been developed for dentists, which potentially can be a part of primary screening. Therefore, the purpose of this study is to establish risk prediction models for dementia and frailty based on OHIP, denture, and clinical characteristics, using a population-based cohort of the Taiwan Longitudinal Study on Aging (TLSA).

## 2. Materials and Methods

### 2.1. Data Source

The TLSA is a population-based prospective cohort study, which was initiated by the Health Promotion Administration, Ministry of Health and Welfare, Taiwan. This survey was firstly conducted in 1989 on adult residents aged 60 or above in non-aboriginal townships of Taiwan [24]. A three-stage systematic random sampling design was used for the selection of an equal probability sample. The first stage sample was drawn from 331 townships, which were stratified by administrative level, three levels of education, and three levels of total fertility rate into 27 strata of roughly equal size [25]. For the second stage, blocks in selected townships, which served as clusters, were selected with probabilities proportional to their size by cumulation of the population. Systemic random sampling was made with the interval of selection equal to the size of each selected township divided by the number of blocks. In the third stage, two eligible respondents were selected by systematic random sampling from each selected block. Data were collected through face-to-face personal interview conducted by trained interviewers. The respondents were later followed every three to four years. Two fresh population samples were selected by the TLSA study group in collaboration with Population Studies Center, University of Michigan in 1996 and 2003 to maintain representativeness of the younger age cohort and to extend representativeness of the sample to the population aged 50 and above. A total of seven surveys were conducted in 1989, 1993, 1996, 1999, 2003, 2007, and 2011 (Wave I–Wave VII), respectively (Supplementary Material Figure S1). In the Wave VII survey, a total of 3727 participants completed interviews, achieving a high response rate of 88.3%. The details and design of the TLSA have been described elsewhere [26].

### 2.2. Study Group Identification, Study Design and Ethical Approval

**Figure 1:**
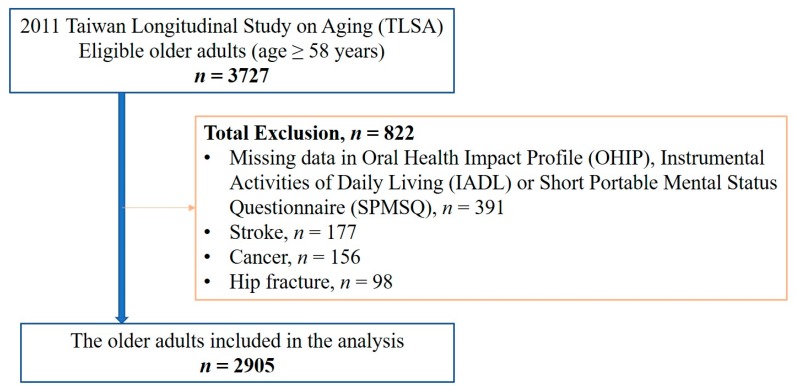
Flow diagram of patient inclusion process.

Since the OHIP-7T was added into the questionnaire since 2011, we analyzed the data collected in the 2011 TLSA database. Of the 3727 eligible older adults aged 58 years and older, 391 participants were excluded due to missing data in the OHIP-7T, Instrumental Activities of Daily Living (IADL) or Short Portable Mental Status Questionnaire (SPMSQ) (Figure 1). Participants with a history of stroke (*n* = 177), cancer (*n* = 156), or hip fracture (*n* = 98) were excluded because these conditions can lead to frailty or dementia [27,28,29]. Ultimately, data from 2905 older adults were utilized in the analysis. The current study was approved by the Institutional Review Board-II of the Kaohsiung Medical University Chung-Ho Memorial Hospital (approval no. KMUHIRB-E(II)-20190124) in 2019.

### 2.3. Research Variables

The gathered subject data comprised age, sex, level of education (uneducated, elementary school, junior high school, senior high school, and college or above), marital status (married, cohabitant, single, divorced, and widowed), self-reported height and weight, smoking, alcohol consumption, and history of major diseases including hypertension, diabetes mellitus (DM), cardiovascular disease, hyperlipidemia, chronic obstructive pulmonary disease (COPD), arthritis, chronic liver disease, and chronic kidney disease (CKD). Body mass index (BMI) was calculated as a person’s weight in kilograms divided by the square of their height in meters (kg/m^2^). BMI was categorized as underweight (BMI < 18.5 kg/m^2^), normal weight (18.5 ≤ BMI < 24.0 kg/m^2^), overweight (24.0 ≤ BMI < 27.0 kg/m^2^), or obese (BMI ≥ 27.0 kg/m^2^), according to the BMI category defined by the Health Promotion Administration, Taiwan [30].

The OHIP-49 has been translated and validated in several countries, and different types of short-form OHIPs for various populations and study purposes have been developed [17,31,32]. In Taiwan, the OHIP-14T and OHIP-7T were developed for use in assessing OHRQoL of older adults, and were employed in national surveys [14,33]. Kuo et al. suggested a conceptual model that OHRQoL, as an important mediator, linked clinical conditions, symptom status, nutritional status, and overall HRQoL [34]. Oral health was assessed in the TLSA using the OHIP-7T and questions relating to dental prosthesis. OHIP-7T was selected from questions in OHIP-49 by controlled regression procedure to have a shorter list of items while maintaining a good reliability and validity [33]. OHIP question responses used a 5-point Likert scale to indicate the frequency of an oral problem over the past 12 months: “very often’’ (score = 4), ‘‘fairly often’’ (score = 3), ‘‘occasionally’’ (score = 2), ‘‘hardly never’’ (score = 1), or ‘‘never’’ (score = 0) [15]. Lower OHIP scores indicated a higher OHRQoL.

### 2.4. Definition of Frailty and Dementia

IADL scale is usually adapted for evaluating older adults with early-stage disease, in order to assess both disease severity and the ability of self-care [35]. In this study, five activities were assessed (preparing meals, shopping, managing money, using the telephone, and taking public transportations independently) using an IADL scale proposed by Lawton and Brody [35]. Respondents who reported that they had difficulty or were unable to carry out the task, or that they had received help or used equipment when performing the task, were coded as having difficulty with the task (1 = yes, 0 = no). IADL disability was defined as a total IADL score ≥ 3 [36]. As observed in a previous study [37], frailty phenotype was also defined by IADL disability in the current study.

Cognitive function was measured by the nine-item SPMSQ, in which correct answers were coded 0 and errors were coded 1, with total scores ranging from 0 to 9 [38]. In this study, the nine questions included were: “where are you located now”; “what is your home address”; “what day, month, and year is it”; “how old are you”; “who are the current and the last presidents”; and “subtract 3 from 20 four consecutive times” [39]. Participants with four or more errors were described as having cognitive impairment, and a previous cohort study supported this cut-off point [40]. In this study, dementia phenotype was defined by cognitive impairment in the SPMSQ.

### 2.5. Statistical Analyses

Descriptive statistics were applied to the demographics and characteristics of the participants. Initially, univariate regression models were used to investigate the association between all variables and frailty or dementia, which were expressed as odds ratios (OR) with 95% confidence interval (CI). We have included all clinical variables including potential confounders in a multivariable logistic regression. Then, the stepwise selection method was applied to the regression to select significant factors [41]. Moreover, risk scores were calculated by stepwise logistic regression to evaluate the association between the clinical covariates shown in Table 1 and frailty or dementia. After the variables were selected, risk scores were computed by dividing the individual odds ratio by the smallest odds ratio in the model and rounding this to the nearest integer. The receiver operating characteristic (ROC) curve and Youden’s index were used to evaluate the diagnostic accuracy of cut-off points for risk scores in the prediction of frailty and dementia [42]. All of the analyses were conducted using SAS version 9.4 (SAS Institute Inc., Cary, NC, USA).

## 3. Results

### 3.1. Characteristics of the Participants

The study sample comprised 2905 participants aged 58 years and above. The mean (±standard deviation) age of the study population was 69.46 (±9.26) years. Of the participants, 50.1% were female, 68.1% were married or cohabitant, and 44.7% were educated to elementary level (44.7%) (Table 1). The mean BMI was 24.34 (±3.43) kg/m^2^; 35.4% were normal weight and 25.4% were overweight. The top three most frequent comorbidities were hypertension (43.0%), diabetes mellitus (16.9%) and cardiovascular disease (16.4%). Approximately 8.2% of participants were defined as frail by IADL scale, and 15.0% had SPMSQ scores suggesting dementia.

1147 (39.5%) participants visited the dentist in the past year. Of the participants, 77.8% had dental prosthesis; 45.6% had fixed dental prosthesis, and 38.3% had removable dental prosthesis. 1696 (58.4%) participants reported at least one of the oral problems in the OHIP-7T, and the mean score of the OHIP-7T was 3.80 (±5.06). The demographics of each question in the OHIP-7T are shown in Table 2.

### 3.2. Development of Risk Models in the Prediction of Frailty and Dementia

We evaluated the association of each variable with frailty and dementia by univariate logistic regression. Each question from OHIP-7T were significantly associated with frailty and dementia (Supplementary Material Table S1). Moreover, the total OHIP-7T score was significantly associated with frailty, with an OR of 1.11 (95% CI, 1.09–1.13; *p* < 0.001) and an area under the curve (AUC) of 0.668. In addition, the total OHIP-7T score was also significantly associated with dementia, with an OR of 1.07 (95% CI, 1.05–1.09; *p* < 0.001) and an AUC of 0.590. The results showed that the higher the OHIP-7T score, the higher the risk of frailty and dementia. However, total OHIP-7T score alone may not be a sufficient screening factor for frailty or dementia due to the low AUC value in each model. Therefore, we combined oral health variables, age, sex, BMI categorization and clinical comorbidities in the risk models for predicting frailty and dementia selected by stepwise logistic regression.

**Figure 2:**
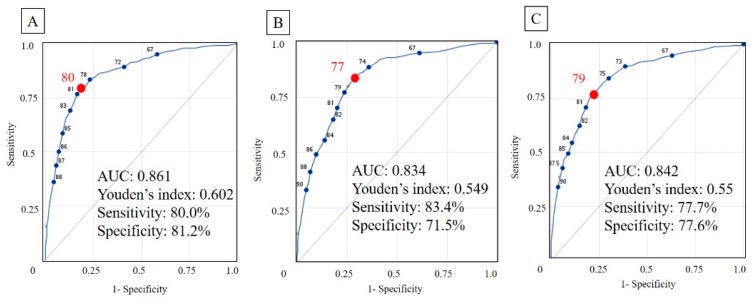
Receiver operating characteristics (ROC) curve of prediction models. The cut-off point was showed as the red dot and number in each ROC curve. (**A**) Model for frailty. (**B**) Model for dementia (**C**) Model for frailty or dementia. AUC, area under the curve.

In model 1 for the prediction of frailty, increased age (adjusted OR [adjOR], 1.17; 95% CI, 1.15–1.20), female sex (adjOR, 1.67; 95% CI, 1.22–2.29), no dental prosthesis (adjOR, 1.61; 95% CI, 1.11–2.35), DM (adjOR, 2.39; 95% CI, 1.68–3.40), CKD (adjOR, 2.59; 95% CI, 1.61–4.17), and an increased OHIP-7T Q3 (“uncomfortable to eat?”) score (adjOR, 1.33; 95% CI, 1.19–1.49) were all significantly associated with frailty (Table 3). The result showed that risk model 1 had a cut-off point of 80 and an AUC of 0.861 (sensitivity, 80.0%; specificity, 81.2%) (Figure 2A).

In model 2 for dementia, increased age (adjOR, 1.12; 95% CI, 1.10–1.13), female sex (adjOR, 2.84; 95% CI, 2.17–3.72), no dental prosthesis (adjOR, 1.45; 1.07–1.97), the score of OHIP-7T_3 (adjOR, 1.17; 95% CI, 1.01–1.37), an increased OHIP-7T Q7 (“taste worse?”) score (adjOR, 1.21; 95% CI, 1.06–1.37), and an inability to self-report height or weight (adjOR, 4.52; 95% CI, 3.52–5.81) were all significantly associated with dementia (Table 3). Model 2 had a cut-off point of 77 and an AUC of 0.834 (sensitivity, 83.4%; specificity, 71.5%) (Figure 2B).

In model 3 for frailty or dementia, increased age (adjOR, 1.14; 95% CI, 1.12–1.15), female sex (adjOR, 2.50; 95% CI, 1.95–3.19), increased OHIP-7T Q3 (adjOR, 1.12; 1.02–1.24) or Q7 (adjOR, 1.22; 95% CI, 1.08–1.38) score, no dental prosthesis (adjOR, 1.51; 95% CI, 1.13–2.01), DM (adjOR, 1.42; 95% CI, 1.07–1.89), CKD (adjOR, 1.56; 95% CI, 1.04–2.33), and an inability to self-report height or weight (adjOR, 3.68; 95% CI, 2.89–4.69) were selected as significant risk factors by stepwise logistic regression (Table 3). Model 3 had a cut-off point of 79 and an AUC of 0.842 (sensitivity, 77.7%; specificity, 77.6%) (Figure 2C).

## 4. Discussion

The current study, using a community representative sample of Taiwan, is the first to develop the easily applicable composite risk scores for the prediction of frailty and dementia. From our results, we found that more frequent oral problems and aging were associated with a higher risk of frailty and dementia with equivalent weighting. We named our risk models collectively as Oral Health for Frailty/Dementia (OHFD) model, which is available online at https://ohfd.riskestimate.online/. Moreover, we found that patients who cannot self-report their height or weight had a statistically higher risk of cognitive impairment. These findings highlight the importance of oral health in older adults and its potential contribution to the early detection of frailty and dementia in dental clinics.

Previous studies have also shown that oral health and function are significantly associated with frailty. The possible mechanisms underlying the associations between oral health and frailty may include nutrition status and oral inflammation [43,44]. Kamdem et al. showed that self-reported oral pain (adjOR, 1.72; 95% CI, 1.17–2.53) and chewing impairment (adjOR, 1.70; 95% CI, 1.07–2.72) were associated with frailty, and demonstrated that this was not solely a result of involuntary weight loss [22]. In addition, patients who rated their oral health as “worse” had a higher likelihood of being frail (OR, 1.56–3.20). Furthermore, cumulative oral health problems (one oral health problem: OR, 2.48; 2 cumulative problems: OR, 2.74; 3 cumulative problems: OR, 3.45) were associated with an increased incidence of frailty [19,45]. The current study showed a consistent result that an individual’s answer to “uncomfortable to eat?” was an independent factor in the prediction of frailty.

Apart from subjective oral problems, objective oral problems are also associated with frailty from previous cross-sectional studies. Especially in older adults aged ≥ 70, Watanabe et al. reported that frail group had significantly fewer present teeth (15.2 ± 5.3 vs. 21.5 ± 7.8), lower occlusal force (317.2 ± 258.0 vs. 509.2 ± 276.7), lower masseter muscle thickness (2.5 ± 0.9 vs. 2.8 ± 1.0), and lower oral diadochokinesis rate than the robust group [18]. Moreover, Semba et al. showed that proportion using dentures were significantly different among non-frail, pre-frail, and frail women, which was 58%, 66%, and 73%, respectively. Women who used dentures and reported difficulty chewing or swallowing had lower five-year survival (Hazard Ratio (HR), 1.43; 95% CI, 1.05–1.97) [46]. In the current study, we demonstrated that having no dental prosthesis was a significant risk factor for frailty and dementia. Additionally, Tanaka et al. reported that accumulated poor oral status such as decreased chewing ability, articulatory oral motor skill, and tongue pressure strongly predicted the incidence of physical frailty (HR, 2.2) and disability (HR, 2.3) among the community-dwelling older adults [47]. Nevertheless, the association between periodontal disease and frailty was still inconclusive [19,46,48]. Therefore, it is likely that early detection of oral health problems and prevention of oral frailty at an earlier stage is essential for healthy aging.

The association between cognitive decline and a low number of teeth was firstly reported by Stein et al. [49]. There has been growing evidence suggesting that poor oral health is associated with cognitive impairment in older adults. The issue highlights the role of the brain-stomatognathic axis in geriatric medicine [50]. Previous studies have demonstrated that suboptimal dentition (<20 teeth) and poorer mastication are associated with a higher risk of dementia or cognitive decline [20,51]. Moreover, the associations between decline in olfactory and gustatory systems and Alzheimer disease was reported [52]. However, a systematic review reported that the association between oral health and cognitive status was inconclusive [53]. The current study showed that having no dental prosthesis and an increased “uncomfortable to eat?” score was significantly associated with cognitive impairment. In addition, an increased “taste worse?” score was also associated with cognitive impairment. It was speculated that gustatory dysfunction may be an early symptom of dementia.

### Limitations

There are some limitations to this study. Firstly, our observational studies cannot examine causal relationships. The current study adopted a cross-sectional design, which cannot establish the association between the risk models and the incidence of frailty and dementia. However, the items in the risk scores were collected in the wave VIII TLSA survey in 2015. Therefore, we would be able to verify the effectiveness of the models after the release of the dataset. Secondly, the mechanism underlying the observed association between oral health and frailty and dementia were not established, so further research is required to elucidate these. Thirdly, although the use of dentures is an adjustable risk factor in older patients, the current study did not demonstrate whether providing suitable dentures could decrease the incidence of frailty and dementia. Fourthly, although we tried our best to evaluate most confounding factors collected in the TLSA database, there are possible unmeasured confounders in the current study. Finally, the current study did not objectively evaluate articulatory oral motor skill, functional performance of the tongue, or masticatory and swallowing function, which may be associated with frailty and dementia.

## 5. Conclusions

Our findings highlight a number of composite risk factors including age, sex, comorbidities and oral health problems in the prediction of frailty and dementia. We developed easily applicable OHFD models which can be used to screen for frailty and dementia in communities or dental clinics.

## Figures and Tables

**Table 1:** Characteristics of the participants of the 2011 Taiwan Longitudinal Study on Aging (TLSA) (*n* = 2905).

Age (Mean ± SD)	69.46 ± 9.26
Sex, *n* (%)	
Male	1426 (49.1)
Female	1479 (50.1)
Height (cm) (mean ± SD) (*n* = 2337)	160.28 ± 7.81
Weight (kg) (mean ± SD) (*n* = 2617)	62.66 ± 10.64
BMI (kg/m^2^) (mean ± SD) (*n* = 2289)	24.34 ± 3.43
Underweight (BMI < 18.5 kg/m^2^), *n* (%)	72 (2.5)
Normal weight (18.5 ≤ BMI < 24 kg/m^2^), *n* (%)	1027 (35.4)
Overweight (24 ≤ BMI < 27 kg/m^2^), *n* (%)	737 (25.4)
Obese (BMI ≥ 27 kg/m^2^), *n* (%)	453 (15.6)
No self-reported height or weight, *n* (%)	616 (21.2)
Alcohol	909 (31.3)
Smoking	410 (14.1)
Marital status, *n* (%)	
Married and Cohabitant	1977 (68.1)
Single and Divorced and Widowed	928 (31.9)
Education, *n* (%)	
Uneducated	578 (19.9)
Elementary school	1299 (44.7)
Junior high school	352 (12.1)
Senior high school	365 (12.6)
College or above	311 (10.7)
Comorbidities, *n* (%)	
Hypertension	1249 (43.0)
Diabetes mellitus	490 (16.9)
Cardiovascular disease	476 (16.4)
Hyperlipidemia	360 (12.4)
Chronic obstructive pulmonary disease	105 (3.6)
Arthritis	415 (14.3)
Chronic liver disease	261 (9.0)
Chronic kidney disease	246 (8.5)
Dental prosthesis, *n* (%)	2261 (77.8)
Fixed dental prosthesis, *n* (%)	1324 (45.6)
Removable dental prosthesis, *n* (%)	1112 (38.3)

**Table 2:** Demographic distribution of the Taiwanese short-form of the Oral Health Impact Profile (OHIP-7T).

	0-Never	1-Hardly Never	2-Occasionally	3-Fairly Often	4-Very Often	Mean ± SD
OHIP-7T Q1: self-conscious? (Q20)	1423 (49.0%)	387 (13.3%)	524 (18.0%)	396 (13.6%)	175 (6.0%)	1.14 ± 1.32
OHIP-7T Q2: interrupt meal? (Q32)	2034 (70.0%)	339 (11.7%)	272 (9.4%)	195 (6.7%)	65 (2.2%)	0.59 ± 1.05
OHIP-7T Q3: uncomfortable to eat? (Q16)	1668 (57.4%)	331 (11.4%)	473 (16.3%)	324 (11.2%)	109 (3.8%)	0.92 ± 1.23
OHIP-7T Q4: concentration affected? (Q37)	2456 (84.5%)	207 (7.1%)	160 (5.5%)	60 (2.1%)	22 (0.8%)	0.27 ± 0.72
OHIP-7T Q5: trouble pronouncing words? (Q2)	2483 (85.5%)	181 (6.2%)	139 (4.8%)	78 (2.7%)	24 (0.8%)	0.27 ± 0.74
OHIP-7T Q6: difficult doing jobs? (Q43)	2645 (91.0%)	134 (4.6%)	72 (2.5%)	37 (1.3%)	17 (0.6%)	0.16 ± 0.57
OHIP-7T Q7: taste worse? (Q6)	2288 (78.8%)	239 (8.2%)	174 (6.0%)	155 (5.3%)	49 (1.7%)	0.43 ± 0.94

(Q) represents the question number from the Oral Health Impact Profile (OHIP)-49 items.

**Table 3:** Prediction of frailty or dementia by the Oral Health for Frailty/Dementia (OHFD) Model.

**Model 1: Prediction of Frailty**				
**Risk Score Items**	**Odds Ratio**	**95% CI**	***p* Value**	**Risk Score Weights ^1^**
Age	1.17	1.15–1.20	<0.001	1
Female	1.67	1.22–2.29	0.001	1
OHIP-7T Q3 score ^2^	1.33	1.19–1.49	<0.001	1
No dental prosthesis	1.61	1.11–2.35	0.01	1
Diabetes mellitus	2.39	1.68–3.40	<0.001	2
Chronic kidney disease	2.59	1.61–4.17	<0.001	2
**Model 2: Prediction of Dementia**				
**Risk Score Items**	**Odds Ratio**	**95% CI**	***p* Value**	**Risk Score Weights ^1^**
Age	1.12	1.10–1.13	<0.001	1
Female	2.84	2.17–3.72	<0.001	3
OHIP-7T Q3 score ^2^	1.17	1.01–1.37	0.04	1
OHIP-7T Q7 score ^3^	1.21	1.06–1.37	0.005	1
No dental prosthesis	1.45	1.07–1.97	0.02	1
Inability to self-report height or weight	4.52	3.52–5.81	<0.001	4
**Model 3: Prediction of Frailty or Dementia**				
**Risk Score Items**	**Odds Ratio**	**95% CI**	***p* Value**	**Risk Score Weights ^1^**
Age	1.14	1.12–1.15	<0.001	1
Female	2.50	1.95–3.19	<0.001	2
OHIP-7T Q3 score ^2^	1.12	1.02–1.24	0.02	1
OHIP-7T Q7 score ^3^	1.22	1.08–1.38	0.001	1
No dental prosthesis	1.51	1.13–2.01	0.005	1
Inability to self-report height or weight	3.68	2.89–4.69	<0.001	3
Diabetes mellitus	1.42	1.07–1.89	0.02	1
Chronic kidney disease	1.56	1.04–2.33	0.03	1

^1^ Odds ratios were calculated by stepwise logistic regression. Risk scores were computed by dividing the individual odds ratio by the smallest odds ratio in the model and rounding this to the nearest integer. ^2^ OHIP-7T Q3: “Are you uncomfortable to eat due to oral problem in recent 12 months?” ^3^ OHIP-7T Q7: “Do you taste worse due to oral problem in recent 12 months?” CI, confidence interval.

## Supplementary Materials

The following are available online at https://www.mdpi.com/1660-4601/17/6/1997/s1, Figure S1: The Establish of Taiwan Longitudinal Study on Aging (TLSA) and Follow-up; Table S1: Univariate logistic regression model in prediction of frailty or dementia.
