# Supplementary material for: Prediction of Frailty and Dementia Using Oral Health Impact Profile from a Population-Based Survey
Source: Int J Environ Res Public Health. 2020 Mar 18;17(6):1997. doi: 10.3390/ijerph17061997 (PMC7143751; doi:10.3390/ijerph17061997)
Supplement: Supplementary file 1 [file ijerph-17-01997-s001.pdf]

Table S1. Univariate logistic regression model in prediction of frailty or dementia.

	Frailty		Dementia	
	**Odds ratio (95% CI)**	**P-value**	**Odds ratio (95% CI)**	**P-value**
OHIP-7T Q1 score: self-conscious?	1.39 (1.26–1.52)	<.001	1.14 (1.06–1.23)	<.001
OHIP-7T Q2 score: interrupt meal?	1.55 (1.40–1.72)	<.001	1.35 (1.24–1.47)	<.001
OHIP-7T Q3 score: uncomfortable to eat?	1.54 (1.40–1.70)	<.001	1.32 (1.22–1.42)	<.001
OHIP-7T Q4 score: concentration affected?	1.63 (1.43–1.86)	<.001	1.29 (1.14–1.45)	<.001
OHIP-7T Q5 score: trouble pronouncing words?	1.59 (1.40–1.80)	<.001	1.27 (1.13–1.43)	<.001
OHIP-7T Q6 score: difficult doing jobs?	1.65 (1.41–1.93)	<.001	1.42 (1.23–1.64)	<.001
OHIP-7T Q7 score: taste worse?	1.55 (1.40–1.72)	<.001	1.44 (1.31–1.57)	<.001
Age	1.16 (1.14–1.19)	<.001	1.13 (1.11–1.14)	<.001
Female	1.50 (1.14–1.97)	.003	3.11 (2.48–3.90)	<.001
Height	0.98 (0.96–1.01)	0.14	0.95 (0.93–0.97)	< .001
Weight	0.98 (0.97–0.99)	.008	0.96 (0.95–0.97)	< .001
Underweight (BMI<18.5 kg/m^2)	2.81 (1.54–5.12)	<.001	1.03 (0.54–1.96)	.94
Normal weight (18.5≦BMI<24 kg/m^2)	0.58 (0.43–0.79)	<.001	0.39 (0.31–0.51)	<.001
Overweight (24≦BMI<27 kg/m^2)	0.48 (0.33–0.70)	<.001	0.29 (0.21–0.40)	<.001
Obese (BMI≧27 kg/m^2)	0.73 (0.49–1.10)	0.13	0.43 (0.30–0.62)	<.001
No self-reported height or weight	3.08 (2.34–4.05)	<.001	8.82 (7.07–11.02)	<.001
Alcohol	0.26 (0.17–0.39)	<.001	0.37 (0.28–0.48)	<.001
Smoking	0.42 (0.25–0.70)	<.001	0.51 (0.35–0.72)	<.001
Married & Cohabitant	0.34 (0.26–0.44)	<.001	0.25 (0.20–0.31)	<.001
Uneducated	3.81 (2.90–5.01)	<.001	10.74 (8.57–13.47)	<.001
Elementary school	0.63 (0.47–0.83)	.001	0.52 (0.42–0.65)	<.001
Junior high school	0.54 (0.32–0.89)	.02	0.24 (0.15–0.41)	<.001
Senior high school	0.58 (0.36–0.94)	.03	0.11 (0.06–0.23)	<.001
College or above	0.46 (0.26–0.82)	.008	0.13 (0.07–0.27)	<.001
Hypertension	1.73 (1.32–2.26)	<.001	1.42 (1.16–1.74)	<.001
Diabetes mellitus	2.08 (1.54–2.81)	<.001	1.11 (0.85–1.45)	.43
Cardiovascular disease	2.01 (1.48–2.73)	<.001	1.46 (1.13–1.88)	.004
Hyperlipidemia	1.53 (1.07–2.19)	.02	0.60 (0.42–0.86)	.005
Chronic obstructive pulmonary disease	1.93 (1.10–3.38)	.02	1.63 (1.01–2.61)	.04
Arthritis	2.20 (1.61–3.00)	<.001	1.55 (1.19–2.02)	.001
Chronic liver disease	1.33 (0.87–2.03)	.18	0.81 (0.55–1.18)	.27
Chronic kidney disease	1.93 (1.31–2.85)	<.001	0.74 (0.50–1.11)	.15
No dental prosthesis	1.01 (0.73–1.38)	.97	0.89 (0.69–1.14)	.35
Fixed dental prosthesis	0.44 (0.33–0.59)	<.001	0.52 (0.42–0.64)	<.001
Removable dental prosthesis	2.23 (1.70–2.91)	<.001	2.04 (1.67–2.51)	<.001

CI, confidence interval

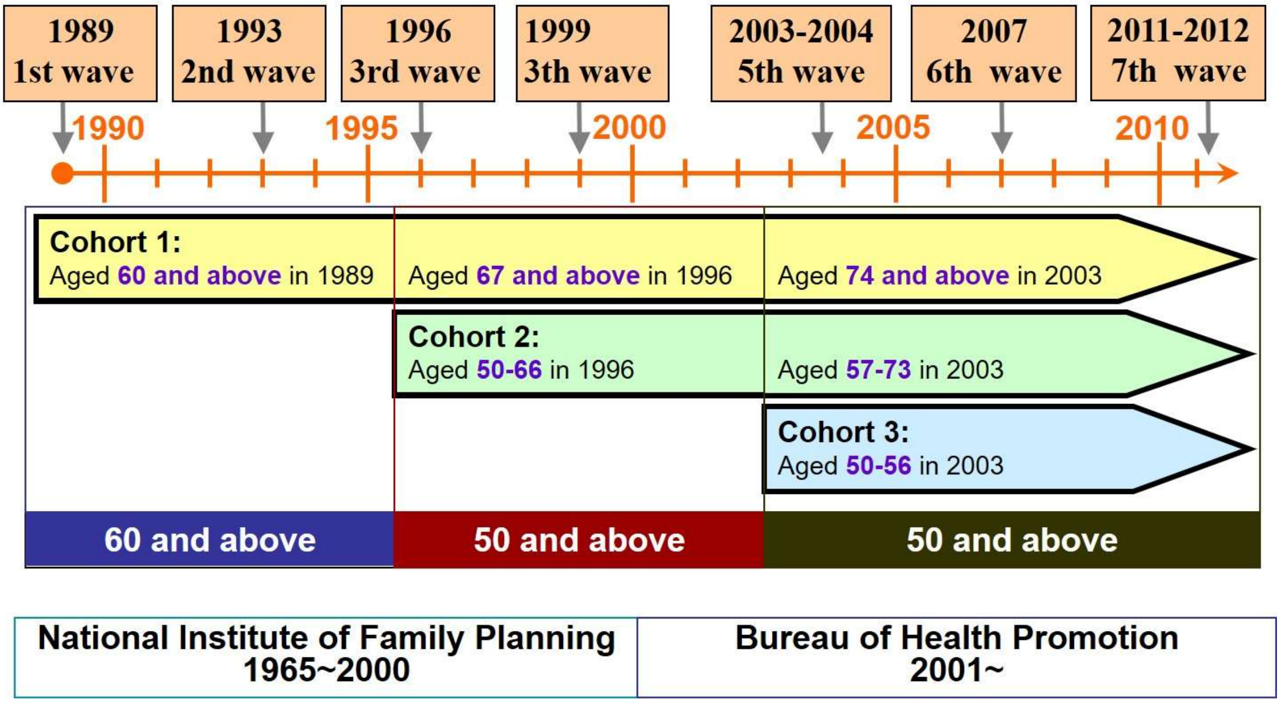

Figure S1. The Establish of Taiwan Longitudinal Study on Aging (TLSA) and Follow-up.
